# Predictive value of remnant-like particle cholesterol in the prediction of long-term AF recurrence after radiofrequency catheter ablation

**DOI:** 10.3389/fcvm.2023.1258125

**Published:** 2023-11-23

**Authors:** Bing Wu, Zichang Liang, Lili Qiu, Jianan Wang, Qianwen Huang, Tingpei Zhuang, Sihan Hong, Meng Bai

**Affiliations:** ^1^ Department of Cardiology, First Hospital of Quanzhou Affiliated to Fujian Medical University, Quanzhou, China; ^2^ Department of General Practice, First Hospital of Putian City, Putian, China

**Keywords:** remnant-like particle cholesterol, radiofrequency catheter ablation, recurrence, atrial fibrillation, prediction

## Abstract

**Objective:**

The relationship between remnant-like particle cholesterol (RLP-C) levels and the progression of atrial fibrillation (AF) is not known. This research aimed to explore the association of RLP-C with long-term AF recurrence events post-radiofrequency catheter ablation (RFCA) of AF.

**Methods:**

In total 320 patients with AF who were subjected to the first RFCA were included in this research. Baseline information and laboratory data of patients were retrospectively collected, and a 1-year follow-up was completed. The follow-up endpoint was defined as an AF recurrence event occurring after 3 months. Afterward, a multivariate Cox regression model was constructed to analyze the risk factors that affect AF recurrence.

**Results:**

AF recurrence occurred in 103 patients (32.2%) within 3–12 months after RFCA. Based on the multivariate Cox regression analysis, Early recurrence (ER) [hazard ratio (HR) =1.57, 95% confidence interval (CI): 1.04–2.36, *P *= 0.032)], coronary artery disease (CAD) (HR = 2.03, 95% CI: 1.22–3.38, *P *= 0.006), left atrium anterior-posterior diameter (LAD) (HR = 1.07, 95% CI: 1.03–1.10, *P *< 0.001), triglyceride (TG) (HR = 1.51, 95% CI: 1.16–1.96, *P *= 0.002), low-density lipoprotein cholesterol (LDL-C) (HR = 0.74, 95% CI: 0.55–0.98, *P* = 0.036), and RLP-C (HR = 0.75 per 0.1 mmol/L increase, 95% CI: 0.68–0.83, *P* < 0.001) were linked to the risk of AF recurrence. Among them, the relationship between RLP-C and AF recurrence was found for the first time. The predictive value of RLP-C for AF recurrence was analyzed utilizing receiver operating characteristic (ROC) curves [area under the curve (AUC) = 0.81, 95% CI: 0.77–0.86, *P* < 0.001]. Subsequently, the optimal threshold value of RLP-C was determined to be 0.645 mmol/L with a sensitivity of 87.4% and a specificity of 63.6% based on the Youden index. Additionally, Kaplan–Meier analysis indicated a lower AF recurrence rate in the >0.645 mmol/L group than in the ≤0.645 mmol/L group (Log-rank *P* < 0.001).

**Conclusion:**

Low levels of RLP-C are associated with a higher risk of AF recurrence post-RFCA, suggesting that RLP-C may be a biomarker that helps to identify long-term AF recurrence.

## 1. Introduction

Atrial fibrillation (AF), a prevalent tachyarrhythmia, continues to result in hospitalization or death from serious complications in approximately 35%–50% of AF patients within 5 years, even with the current guideline approach to disease management (1). Radiofrequency catheter ablation (RFCA), based on the circumferential pulmonary vein isolation technique, has been recommended by AF management guidelines (2) as the first-line treatment for radical treatment of AF. However, postoperative recurrent events pose a burden to patients and clinicians. The long-term AF recurrence post-RFCA rate can reach 30%–50% (3, 4). Individuals with persistent AF have a higher degree of atrial fibrosis. Despite the availability of modified stroma-based ablation techniques, the AF recurrence post-RFCA rate remains high. A recent investigation found a postoperative recurrence rate of 35.2% in persistent AF with the “2C3l” procedure (5). Therefore, the identification of risk factors affecting prognosis and active intervention is important to enhance the outcome of RFCA and decrease the incidence of AF recurrence.

As research continues, some progress has been made in addressing potential risk factors for the occurrence and AF recurrence (6–8). Individuals with combined coronary artery disease (CAD) have a higher incidence of AF (9, 10) by various mechanisms, including inflammation, atrial stretch, atrial ischemia, and autonomic and hormonal activation. TC and LDL-C, as risk factors for CAD (11), should theoretically be positively associated with the onset and progression of AF. However, the existing studies do not support this view. The levels of cholesterols, including TC and LDL-C, were negatively linked to the long-term AF recurrence rate post-RFCA (12, 13). Evidence from several well-designed studies (14–16) confirms that high levels of TC, LDL-C, and VLDL-C are protective factors for new-onset AF. The paradoxical inverse relationship between the above cholesterol levels and AF is known as the cholesterol paradox phenomenon (9). In addition, the same consistent results were observed in studies correlating HDL-C with the occurrence of AF recurrence (17, 18).

Remnant-like particle cholesterol (RLP-C), another important member of the cholesterol family, is of great interest as an independent risk factor for atherosclerotic cardiovascular disease (ASCVD) (11, 19). Even in individuals with normal LDL-C, RLP-C remains a key causative factor in the development of ASCVD events (19–21). As a crucial cholesterol indicator, its association with long-term AF recurrence post-RFCA, like other family members, is not known. Hence, this research sought to examine the correlation between RLP-C and long-term AF recurrence post-RFCA and its predictive value., thereby further clarifying the relationship between cholesterol levels and long-term AF recurrence post-RFCA.

## 2. Material and methods

### 2.1. Research subjects

In total, 364 individuals were selected for this retrospective cohort study as per corresponding diagnostic criteria and were diagnosed as AF at the First Hospital of Quanzhou Affiliated to Fujian Medical University, between January 2019 and June 2022. In addition, all individuals met the following inclusion criteria: (a) All of these individuals met the guideline-recommended indications for RFCA (2). (b) All individuals underwent RFCA for the first time and were successfully converted to sinus rhythm by ablation. (c) These patients underwent preoperative collection of basic information as well as electrocardiography, echocardiography, and laboratory tests. Patients with incomplete baseline information, unavailable follow-up information, and taking oral lipid-lowering drugs before the collection of fasting blood specimens were not included in this research. Ultimately, 320 individuals were selected for follow-up analysis. This research was approved by the Ethics Review Committee of First Hospital of Quanzhou Affiliated to Fujian Medical University (approval No.: 2023K031). The study was retrospective in design and therefore exempted from informed consent. Additionally, all procedures in this research were executed according to the Declaration of Helsinki and all relevant guidelines and regulations.

### 2.2. Baseline information

Baseline characteristics of individuals comprising age, gender, histories of alcohol abuse and smoking, body mass index (BMI), type of AF, and histories of hypertension, diabetes mellitus, coronary artery disease, and hyperthyroidism were collected at the time of admission.

### 2.3. Blood lipid level measurement

Fasting blood samples were acquired from individuals early in the morning before ablation on the second day after their admission to the hospital. A set of biochemical markers, encompassing total cholesterol (TC), low-density lipoprotein cholesterol (LDL-C), very low-density lipoprotein cholesterol (VLDL-C), high-density lipoprotein cholesterol (HDL-C), and triglycerides (TG) were assessed. There is no consensus on the measurement of RLP-C because RLP is difficult to separate from its precursors. The available methods include the formula and direct measurement techniques (including direct automated analysis, immunoseparation, ultracentrifugation, etc.) (22). The direct measurement technique has not been recommended in clinical practice due to high equipment requirements, operational difficulties, and high costs. In contrast, the formula technique has gained widespread applications in clinical studies due to its convenience, rapidity, and economical efficiency (19–21). The RLP-C derived by the formula technique can accurately reflect the actual measurement level of RLP-C (23). Therefore, the formula technique was used for RLP-C measurement in this research: RLP-C = TC-(LDL-C + HDL-C).

### 2.4. Cardiac ultrasound indicators

Transthoracic echocardiography was performed by an experienced sonographer and recorded with a nationally approved diagnostic echocardiography system (Philips IE Elite, Philips Ultrasound, Inc., Bothell, WA, USA) with a probe frequency of 2.0–4.0 MHz. Specifically, the individual was placed in a left lateral position to fully expose the thorax. Subsequently, cardiac ultrasound data were collected in parasternal short and long-axis views, as well as apical four-chamber and two-chamber views in sequence. The left atrium anterior-posterior diameter (LAD) was measured at the end of the left ventricular systole. Finally, the left ventricular ejection fraction (LVEF) was measured using a dual-plane method.

### 2.5. RFCA

Three-dimensional reconstruction of the left atrium and bilateral pulmonary ostium venosum sites was performed with the aid of an electroanatomical landmarking system (Carto3, Biosense Webster, Diamond Bar, CA, USA). Circumferential pulmonary vein ablation (CPVA) was performed in individuals with paroxysmal AF utilizing an adjustable curved radiofrequency ablation catheter (Thermcool ST, Biosense Webster, Diamond Bar, CA, USA) (target temperature 43°C, maximum power 45 W). Individuals with persistent AF underwent CPVA combined with top-line as well as bottom-line ablation of the left atrium. Additionally, other linear ablations, such as mitral isthmus addition, tricuspid isthmus addition, and linear ablation of the upferior vena cava were added at the discretion of the operator, depending on whether atrial arrhythmias were induced. Details of the CPVA procedure have been illustrated in an expert consensus statement (24). The endpoint of successful ablation is defined as the completion of the circumferential ablation trail of the pulmonary veins, as well as other additional ablation lines while achieving a bidirectional conduction block on both sides of the ablation line.

### 2.6. Postoperative follow-up

The 1-year follow-up data of individuals with AF after surgery were recorded, in which 3-month recurrence was defined as the endpoint for follow-up. The initial follow-up visit was scheduled on day 7 after discharge. Subsequent follow-up visits were then recommended every 3 months thereafter. All individuals were recommended to undergo immediate reexamination if they developed symptoms similar to AF. Patients who were not followed up promptly were contacted by telephone to record the onset of arrhythmia symptoms including palpitations, chest distress, and fatigue, and were instructed to seek prompt medical care. A long-term AF recurrence was defined as the presence of atrial tachyarrhythmia that last for more than 30 s (recorded by ECG or 24-h-Holter) after 3 months of ablation. In our practice, in order to improve the detection rate of atrial tachyarrhythmia, we routinely carry out 12-lead ECG on patients undergoing review, and further 24-h-Holter are performed on patients in whom atrial tachyarrhythmia is not recorded. A recurrence within 3 months is referred to as a blanket recurrence or early recurrence (ER). In the past, AF recurrence within 3 months was considered to be a recurrence without clinical significance, and it was thought that ER was associated with inflammation and edema of tissues and cells after RFCA. However, recent research evidence suggests a higher risk of long-term recurrence among patients with early recurrence (3). Therefore, it was used as an important covariate in the follow-up analysis. Postoperative antiarrhythmic drug (AAD) administration and anticoagulation were maintained for at least 3 months. After 3 months, AADs were discontinued in the absence of atrial tachyarrhythmia recurrence. Furthermore, anticoagulants were discontinued or reduced according to the CHA_2_DS_2_-VASc score.

### 2.7. Statistical analysis

The mean ± standard deviation was utilized for expressing normally distributed data, and the t-test was employed for comparisons in this case. Non-normally distributed data, on the other hand, were expressed as the median and interquartile range (IQR), and comparisons were made utilizing the Mann–Whitney *U*-test in such cases. Percentages were utilized for the expression of categorical variables, and comparative assessment was done via the *χ*^2^ test. Univariate Cox regression analysis was conducted to identify risk factors for long-term AF recurrence and calculate hazard ratios (HRs) and 95% confidence intervals (95% CIs). Independent variables with a *P* < 0.1, as well as lipid factors, gender and age, were incorporated in the multivariate Cox regression analysis. Backward partial likelihood estimation was utilized for eliminating collinearity and constructing a multivariate regression model. The link between RLP-C levels and AF recurrence was analyzed using these models, followed by the calculation of HRs and 95% CIs. To evaluate the robustness of the findings, subgroup analyses were conducted for gender, ER, and type of AF, and forest plots were generated. ROC curves were plotted to determine the predictive value of RLP-C in long-term AF recurrence after RFCA and calculate the sensitivity and specificity. Moreover, the Youden index was employed to evaluate the optimal cut-off point. The study subjects were then categorized into two groups: the high RLP-C level group and the low RLP-C level group, as per the aforementioned cut-off point. Kaplan–Meier curves were then generated. Subsequently, the log-rank test was performed to assess any significant variation between both groups. All the analyses described above were executed utilizing SPSS 23.00 (SPSS, Inc., Chicago, IL, USA) and GraphPad Prism 9.0.2 for Windows (GraphPad Software, San Diego, CA, USA). A *P* < 0.05 denoted the statistical significance level.

## 3. Results

### 3.1. Inclusion, exclusion, and grouping processes

**Figure 1:**
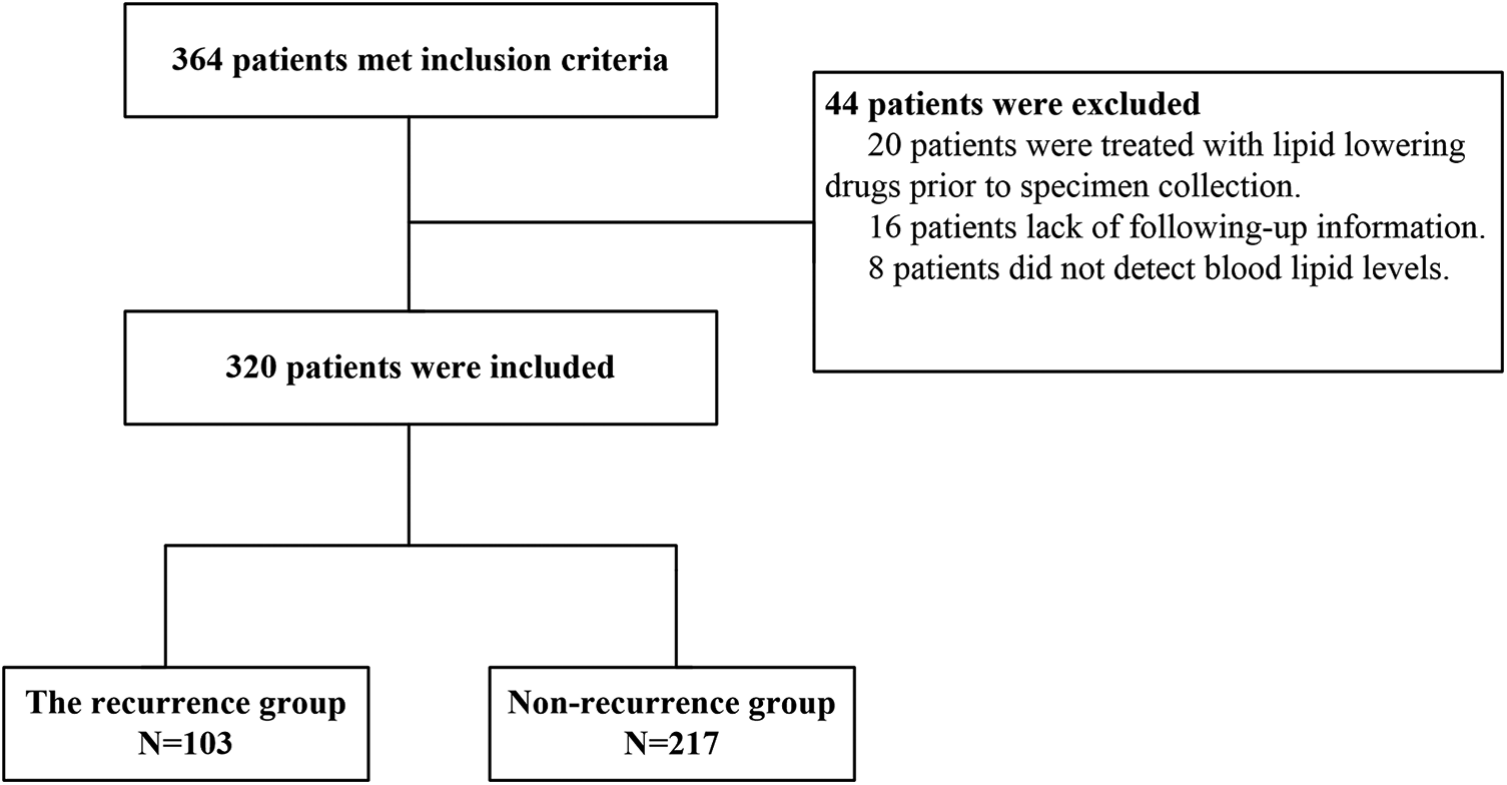
Selection process of research subjects.

Figure 1 illustrates that 364 individuals who satisfied the diagnostic criteria for AF and underwent their first RFCA procedure between January 2019 and June 2022, were initially enrolled in the research. Subsequently, 44 individuals were excluded, including 20 individuals taking lipid-lowering drugs before blood specimen collection, 16 individuals with no follow-up information, and 8 individuals whose lipid levels were not measured before the procedure. As per the inclusion and exclusion criteria, 320 individuals were selected. Among them, 103 individuals were classified into the recurrence group, while 217 individuals were categorized into the non-recurrence group. Recurrence herein was defined as long-term recurrence occurring after 3 months.

### 3.2. Baseline data of individuals in the recurrence and non-recurrence groups

During the 12-month follow-up, 103 individuals experienced recurrence. As presented in Table 1, the recurrence group was older (65.0 vs. 62.0; *P *= 0.046) and showed a greater proportion of ER (37.9% vs. 14.3%; *P* < 0.001) in comparison to the non-recurrence group. The recurrence group had a greater proportion of diabetes mellitus (30.0% vs. 20.7%; *P* < 0.001) and CAD (18.9% vs. 6.9%; *P* < 0.001) and longer LAD (43.0 vs. 37.0; *P* < 0.001). However, the proportion of paroxysmal AF was reduced in the recurrence group in comparison to that in the non-recurrence group (47.6% vs. 74.7%; *P* < 0.001). In addition, the levels of TC (3.9 vs. 5.1; *P *< 0.001), LDL-C (2.4 vs. 3.1; *P* < 0.001), HDL-C (1.0 vs. 1.1; *P* < 0.001), and RLP-C (0.4 vs. 0.7; *P* < 0.001) were decreased in the recurrence group in comparison to the non-recurrence group. Moreover, no statistically significant variations in age, gender, BMI, smoking, alcohol abuse, hypertension, hyperthyroidism, LVEF, and TG were noted across the two groups in this research (*P* > 0.05).

**Table 1:** Comparison of baseline information between the two groups of patients.

Baseline characteristics	All	Recurrent group	Non-recurrent group	*P*-Value
	*N* = 320	*N* = 103	*N* = 217	
Age, median (IQR), y	63.0 (56.0, 69.0)	65.0 (58.0, 70.0)	62.0 (55.0, 68.0)	0.046
Female, *n* (%)	132 (41.3%)	40 (38.9%)	92 (42.4%)	0.545
BMI, mean ± SD, kg/m^2^	25.0 ± 3.2	25.5 ± 3.5	24.7 ± 3.1	0.056
Smoking, *n* (%)	65 (20.3%)	16 (15.5%)	49 (22.6%)	0.143
Alcohol abuse, *n* (%)	22 (6.9%)	7 (6.8%)	15 (6.9%)	0.969
Paroxysmal AF, *n* (%)	211 (65.9%)	49 (47.6%)	162 (74.7%)	<0.001
ER, *n* (%)	70 (21.9%)	39 (37.9%)	31 (14.3%)	<0.001
Previous history				
Hypertension, *n* (%)	165 (51.6%)	56 (54.4%)	99 (45.6%)	0.144
Diabetes, *n* (%)	76 (23.8%)	31 (30.0%)	45 (20.7%)	0.066
Hyperthyroidism, *n* (%)	15 (4.7%)	5 (4.8%)	10 (4.6%)	0.922
CAD, *n* (%)	34 (10.6%)	19 (18.4%)	15 (6.9%)	0.002
Cardiac function				
LAD, median (IQR), mm	38.0 (35.0, 44.0)	43.0 (38.0, 46.0)	37.0 (34.0, 41.0)	<0.001
LVEF, median (IQR), %	63.0 (60.0, 67.0)	62.0 (57.0, 68.0)	63.0 (60.0, 67.0)	0.401
Lipid level				
TC, median (IQR), mmol/L	4.7 (3.9, 5.6)	3.9 (3.2, 4.5)	5.1 (4.4, 5.8)	<0.001
TG, median (IQR), mmol/L	1.3 (0.9, 1.8)	1.3 (0.8, 2.0)	1.2 (1.0, 1.7)	0.402
LDL-C, median (IQR), mmol/L	2.9 (2.3, 3.5)	2.4 (1.9, 3.0)	3.1 (2.6, 3.8)	<0.001
HDL-C, median (IQR), mmol/L	1.1 (0.9, 1.3)	1.0 (0.8, 1.2)	1.1 (1.0, 1.4)	<0.001
RLP-C, median (IQR), mmol/L	0.6 (0.4, 0.8)	0.4 (0.3, 0.6)	0.7 (0.5, 0.9)	<0.001

BMI, body mass index; ER, early recurrence; Paroxysmal AF, paroxysmal atrial fibrillation; CAD, coronary artery disease; LAD, left atrium diameter; LVEF, left ventricular ejection fraction; TC, total cholesterol; TG, triglyceride; LDL-C, low-density lipoprotein cholesterol; HDL-C, high-density lipoprotein cholesterol; RLP-C, remnant-like particle cholesterol.

### 3.3. Predictors of long-term AF recurrence

As presented in Table 2, in the univariate Cox regression analysis, statistically significant predictors for AF recurrence included paroxysmal AF (HR = 0.41, 95% CI: 0.28–0.60*, P* < 0.001), ER (HR = 2.58, 95% CI: 1.72–3.83, *P *< 0.001), CAD (HR = 2.46, 95% CI: 1.49–4.05, *P* < 0.001), LAD (HR = 1.11, 95% CI: 1.08–1.14*, P* < 0.001), LVEF (HR = 0.98, 95% CI: 0.96–0.99, *P* = 0.007), TC (HR = 0.93, 95% CI: 0.91–0.95, *P* < 0.001), TG (HR = 1.34, 95% CI: 1.01–1.79, *P* = 0.049), LDL-C (HR = 0.43, 95% CI: 0.33–0.56, *P* < 0.001), and every 0.1 mmol/L rise in HDL-C (HR = 0.88, 95% CI: 0.80–0.93, *P* < 0.001) and RLP-C (HR = 0.69, 95% CI: 0.64–0.76, *P* < 0.001).

**Table 2:** Factors influencing the long-term AF recurrence.

Variables	Univariate			Mulitvariate^†^		
	HR	95% CI	*P*-Value	HR	95% CI	*P*-Value
Age (y)	1.02	0.99–1.04	0.088	1.01	0.98–1.02	0.957
Female, *n* (%)	1.14	0.77–1.70	0.517	1.11	0.73–1.67	0.628
BMI (kg/m^2^)	1.05	0.99–1.11	0.080	0.99	0.94–1.05	0.822
Smoking, *n* (%)	0.69	0.40–1.17	0.170			
Alcohol abuse, *n* (%)	1.03	0.48–2.22	0.939			
Paroxysmal AF, *n* (%)	0.41	0.28–0.60	<0.001	1.04	0.65–1.68	0.865
ER, *n* (%)	2.58	1.72–3.83	<0.001	1.57	1.04–2.36	0.032
Hypertension, *n* (%)	1.32	0.89–1.94	0.165			
Diabetes, *n* (%)	1.52	0.99–2.32	0.051	1.03	0.66–1.61	0.902
Hyperthyroidism, *n* (%)	1.05	0.43–2.57	0.919			
CAD, *n* (%)	2.46	1.49–4.05	<0.001	2.03	1.22–3.38	0.006
LAD (mm)	1.11	1.08–1.14	<0.001	1.07	1.03–1.10	<0.001
LVEF (%)	0.98	0.96–0.99	0.007	1.00	0.98–1.02	0.968
TC (mmol/L)	0.93	0.91–0.95	<0.001	1.01	0.88–1.16	0.910
TG (mmol/L)	1.34	1.01–1.79	0.049	1.51	1.16–1.96	0.002
LDL-C (mmol/L)	0.43	0.33–0.56	<0.001	0.74	0.55–0.98	0.036
HDL-C (mmol/L)	0.88	0.80–0.93	<0.001	0.97	0.89–1.05	0.437
Per 0.1 mmol/L						
RLP-C (mmol/L)	0.69	0.64–0.76	<0.001	0.75	0.68–0.83	<0.001
Per 0.1 mmol/L						

^†^ Multivariate regression model: adjusted for age, gender, BMI, diabetes, atrial fibrillation types, ER, CAD, LAD, LVEF, TC, TG, LDL-C, HDL-C, RLP-C.

The variables with *P* < 0.1 in the above univariate Cox regression analysis and gender underwent a multivariate Cox regression model and were analyzed by employing a backward stepwise screening technique. The statistically significant independent predictors in the final analysis included ER (HR = 1.57, 95% CI: 1.04–2.36, *P *= 0.032), CAD (HR = 2.03, 95% CI: 1.22–3.38, *P *= 0.006), LAD (HR = 1.07, 95% CI: 1.03–1.10, *P *< 0.001), TG (HR = 1.51, 95% CI: 1.16–1.96, *P *= 0.002), LDL-C (HR = 0.74, 95% CI: 0.55–0.98, *P *= 0.036) and every 0.1 mmol/L rise in RLP-C (HR = 0.75, 95% CI: 0.68–0.83, *P *< 0.001).

### 3.4. Subgroup analysis of RLP-C

**Figure 2:**
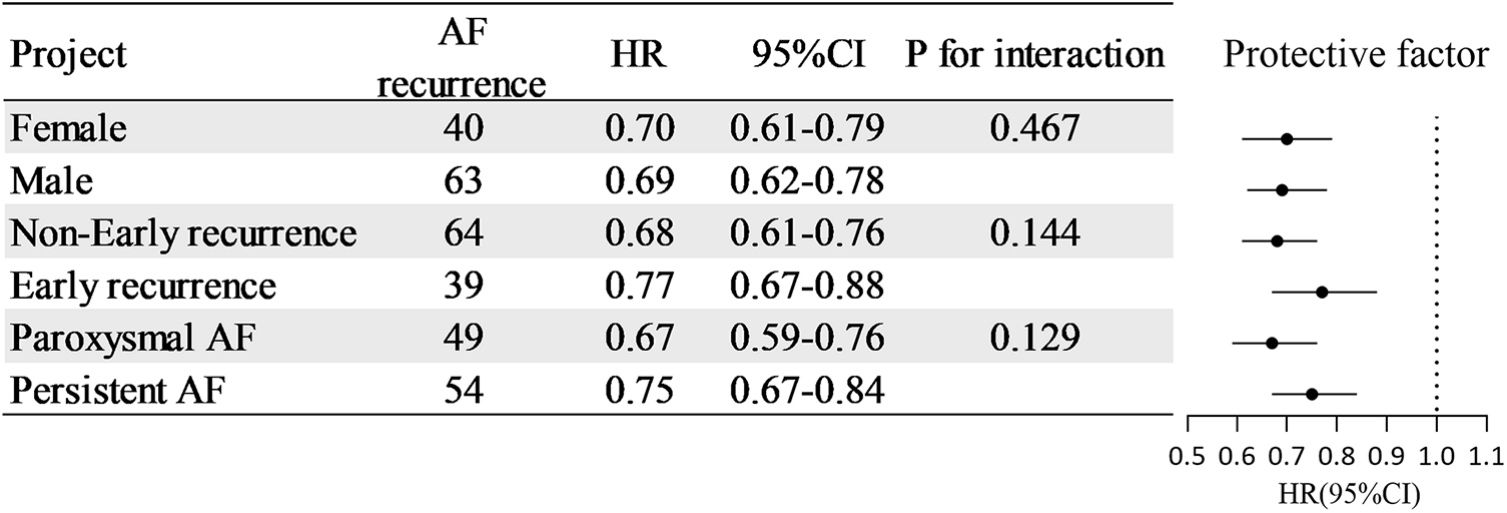
Subgroup analysis of RLP-C as a predictor of long-term AF recurrence.

To assess the stability of the results, validation was performed in three subgroups. As presented in Figure 2, RLP-C exhibited an inverse relation with AF recurrence post-RFCA in all subgroups in terms of male (HR = 0.69, 95% CI: 0.62–0.78, *P *< 0.001), female (HR = 0.70, 95% CI: 0.61–0.79, *P *< 0.001), ER (HR = 0.77, 95% CI: 0.67–0.88, *P *< 0.001), non-ER (HR = 0.68 95% CI: 0.61–0.76, *P *< 0.001), paroxysmal AF (HR = 0.67, 95% CI: 0.59–0.76, *P *< 0.001), and persistent AF (HR = 0.75, 95% CI: 0.67–0.84, *P *< 0.001). In addition, no considerable variations were observed at the respective subgroup levels of sex, presence of ER, and AF type, with *P*-values for the interaction of 0.467, 0.144, and 0.129, respectively.

### 3.5. ROC curve of RLP-C

**Figure 3:**
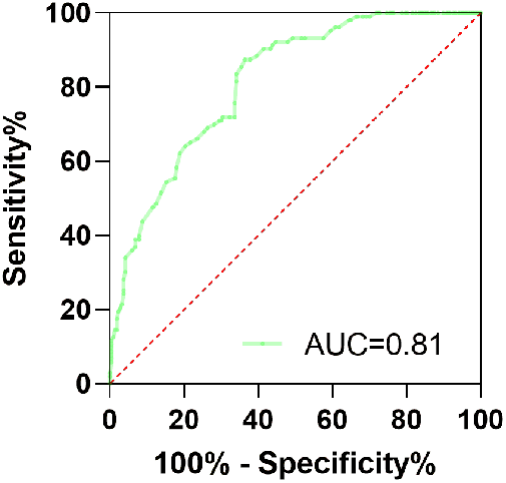
ROC curves for RLP-C predicting long-term AF recurrence.

ROC analysis was executed to further assess the predictive value of RLP-C for AF recurrence. As presented in Figure 3, the area under the curve (AUC) value of RLP-C was 0.81 (95% CI: 0.77–0.86, *P* < 0.001), with the maximum Youden index of 0.51, the optimal threshold value of RLP-C of 0.645 mmol/L, sensitivity of 87.4%, and specificity of 63.6%.

### 3.6. Survival analysis

**Figure 4:**
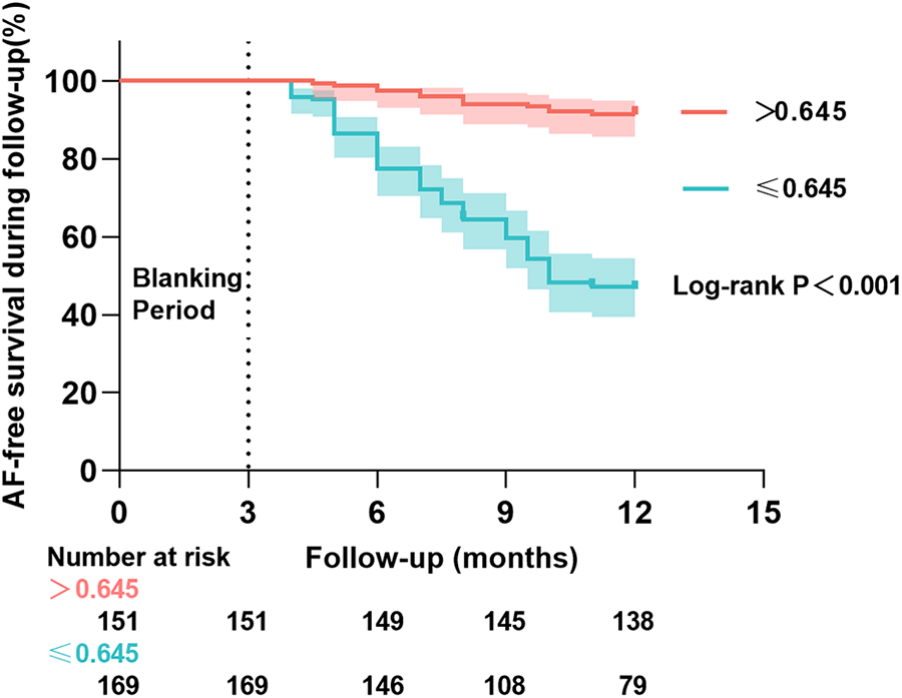
Kaplan–Meier cumulative AF-free curves for long-term AF recurrence events at different RLP-C levels.

The 320 patients were regrouped into two groups (≤0.645 mmol/L group and >0.645 mmol/L group) according to the optimal RLP-C threshold value of 0.645 mmol/L, and Kaplan–Meier survival curves were plotted (Figure 4). A total of 103 patients (32.2%) experienced recurrence during the follow-up period. Of these, 90 (53.3%) recurrences occurred at the end of follow-up in the ≤0.645 mmol/L group and 13 (8.6%) occurred in the >0.645 mmol/L group. According to the Log-rank test, the AF recurrence rate was reduced in the >0.645 mmol/L group than in the ≤0.645 mmol/L group (Log-rank *P* < 0.001). After adjusting for ER, CAD, LAD and LDL-C, multivariate Cox regression analysis showed that lower recurrence rates of AF in the high RLP-C level (>0.645 mmol/L) group compared with ≤0.645 mmol/L group (HR = 0.21, 95% CI: 0.11–0.39, *P* < 0.001).

## 4. Discussion

This research reported the factors affecting the long-term recurrence after AF. The roles of ER, CAD, LAD, and LDL-C were in line with the findings of studies conducted in the past (6–8). To our knowledge, this is the pioneering research to illustrate a negative association of RLP-C with long-term AF recurrence. Even after accounting for potential confounding factors, this relationship remained statistically significant. Furthermore, consistent findings were observed across subgroup analyses Further analysis revealed a good predictive value of RLP-C in terms of long-term AF recurrence postoperatively. However, higher-quality prospective studies are still needed for validation.

The complex pathogenesis of AF mainly involves atrial remodeling, oxidative stress, and inflammatory response (25). Current studies have classified the influencing factors involved in these mechanisms into modifiable and non-modifiable factors. Cholesterol, as one of the common modifiable factors, is involved in normal physiological functions and can be involved in the development of several diseases through abnormal accumulation. The correlation between cholesterol and AF was discovered as early as 1997 by Psaty et al. (26). They found that higher cholesterol levels were linked to a reduced risk of new-onset AF. After a similar finding, Annour et al. (27) proposed the “cholesterol paradox phenomenon”. Since then, this phenomenon has been confirmed in many primary and secondary studies (12–17). The inverse relationship between LDL-C and RLP-C and AF recurrence post-RFCA in the present study also supports this phenomenon.

Rhythm control, especially early rhythm control, can be more beneficial for AF patients (28). In terms of rhythm control, the application of RFCA for AF is more advantageous compared to the treatment of AADs (29). Despite the continuous advances in ablation strategies, recurrent events are still unavoidable. In recent years, a well-designed prospective cohort study (12) found for the first time an independent association between LDL-C and AF recurrence post-RFCA (HR = 0.61, 95% CI: 0.44–0.87, *P* = 0.005), which aligns with the outcomes of the current investigation. The above research also found a negative association between TC and AF recurrence (HR = 0.59, 95% CI: 0.43–0.81, *P *= 0.001). Unlike the aforementioned studies, the current investigation discovered that TC was not related to AF recurrence post-RFCA after controlling for confounding factors, including RLP-C. This difference may be related to RLP-C. The correlation between HDL-C and AF recurrence was not found by the research team and was in line with the outcomes of Shang Y et al. (3) and Li ZZ et al. (13). Furthermore, Canpolat et al. (30) performed cryoballoon ablation in 402 individuals with AF with a mean follow-up of 20.6 months and found that levels of HDL-C were considerably reduced in the recurrence group in comparison to the non-recurrence group (0. 93 mmol/L vs. 1.14 mmol/L, *P* < 0.001). The reasons behind the controversy are unclear and need to be explored in depth by high-quality studies.

Statins can reduce AF recurrence by improving atrial remodeling and anti-inflammatory effects while lowering cholesterol levels (31). In contrast, in a double-blind randomized controlled study (32), 125 individuals with AF to be treated with RFCA were assigned randomly to the atorvastatin (80 mg/day) and the placebo categories and received oral drug therapy from postoperative day 1 until 3 months postoperatively. The difference in AF recurrence rates across the atorvastatin and placebo categories was not statistically significant (5% vs. 6.5%, *P* = 0.37). A similar inference was obtained from a meta-analysis involving nine studies (33). Thus, the role of statins in preventing AF recurrence was not recognized, suggesting a non-benefit of cholesterol reduction on AF recurrence, indirectly proving our point. The use of statins in the perioperative period in patients with AF during clinical practice deserves careful consideration.

RLP is a relatively apoE-rich lipoprotein particle formed after triglyceride-rich lipoproteins (TGRLs) are metabolized by lipoprotein esterase (LPL) (34). RLP is free from oxidation. Therefore, it is often taken up by macrophages and smooth muscle cells before LDL to initiate the inflammatory response. The cholesterol component of RLP is known as RLP-C (22). Many high-quality studies have confirmed RLP-C as a residual risk factor for ASCVD (19–21). The association between RLP-C and postoperative recurrence of AF was first elaborated in this research. Previously, the association between the two was not clear. This negative association persisted after controlling for confounding factors including other cholesterol components, for each 0.1 mmol/L rise in RLP-C (HR = 0.75, 95% CI: 0.68–0.83, *P* < 0.001). However, the mechanism underlying the link between the two is unclear. Differences in lipid levels exist between genders (35). Therefore, in order to evaluate the relationship between RLP-C and AF recurrence in different genders, we performed a subgroup analysis. The results showed that the inverse relationship was consistent in both male and female, and the extent of the association was not statistically different between the sexes. In addition, the impact of AF type and ER on the findings was further analyzed, and no meaningful results were observed.

Studies on the mechanisms between cholesterol and AF are still limited. To date, the mechanisms of the two include the following: (a) Cholesterol affects cardiac cell membrane stability by influencing ion channels and the sensitivity of volume-regulated anion currents to osmotic gradients (36). Low levels of cholesterol reduce cell membrane stability thereby promoting the formation of arrhythmogenic potentials. (b) The link between cholesterol levels and AF may also be related to inflammation (37). During inflammation, levels of TC, LDL-C, and HDL-C levels are reduced while that of TG are increased (38). Current research illustrated a positive association of TG with AF recurrence after controlling for confounding factors (HR = 1.51, 95% CI: 1.16–1.96, *P *= 0.002). Hence, reduced cholesterol levels as well as elevated or reactive TG levels may act as potential inflammatory processes leading to the development of AF. (c) Lipoprotein particle subtypes of different sizes may be the driving force for the negative association of cholesterol with AF (39). Past studies have found smaller lipoprotein particles, higher levels of oxidation, glycosylation, and TG, and reduced cholesterol levels in individuals with AF (40, 41). (4) Individuals with a history of hyperthyroidism have a considerably greater number of PV ectopic foci and an increased rate of AF recurrence following a single procedure (42). Additionally, hyperthyroidism can lower cholesterol levels, ultimately leading to the cholesterol paradox phenomenon. However, an association between hyperthyroidism and AF recurrence was not observed in this research (HR = 1.05, 95% CI: 0.43–2.57, *P* = 0.919).

Whether the above mechanisms can fully explain the relationship between RLP-C and AF recurrence post-RFCA requires further investigation. Notably, RLP-C was noted to have a predictive value for AF recurrence post-RFCA (AUC = 0.81, 95% CI: 0.77–0.86, *P *< 0.001). The utilization of this indicator can assist in promptly identifying individuals at high risk for recurrence, enabling clinicians to implement appropriate measures and implement closer cardiac monitoring to minimize recurrence. The optimal threshold for RLP-C according to the Youden index analysis is 0.645 mmol/L. It is essential to exercise caution when extrapolating the conclusions of this research due to the exclusion of individuals who were taking oral lipid-lowering drugs. Future investigations should focus on determining effective strategies for managing cholesterol levels in individuals with endogenous hypocholesterolemic AF and those with AF combined with CAD to reduce the risk of postoperative recurrence.

## 5. Limitations

The retrospective design of this research resulted in uncontrolled biased information. Therefore, only a correlation between RLP-C and AF recurrence after surgery can be indicated, and the causal relationship cannot be elucidated. Additionally, the sample size of this research was relatively small, which may have an impact on the statistical power and efficacy of the tests conducted. Moreover, this study only measured baseline lipid levels before ablation, whereas the lipid levels during follow-up and at the time of recurrence are unknown. Thus, the association between changes in lipid levels and AF recurrence post-RFCA could not be clarified. In this study, the error between RLP-C and true levels was measured by the formula method. The diagnosis of AF recurrence relied on the assessment of symptoms and electrocardiographic findings. Symptoms play a dominant role in prompting people to seek medical care. Consequently, the recurrence of asymptomatic individuals is not available in the first place, which may lead to errors in follow-up information.

## 6. Conclusion

To conclude, this research represents the first identification of an inverse relationship between RLP-C and long-term AF recurrence following RFCA. These findings align with the cholesterol paradox phenomenon. Moreover, RLP-C was found to have a promising predictive value for AF recurrence post-RFCA events, indicating its potential utility in the context of individualized medicine. However, it is important to acknowledge that the retrospective design of this research introduces inherent limitations. Therefore, future studies with higher methodological quality need to be conducted to further investigate the relationship between RLP-C and AF recurrence. These studies should aim to elucidate the precise mechanisms underlying this relationship.

## Data Availability

The raw data supporting the conclusions of this article will be made available by the authors, without undue reservation.
